# 6mA-RicePred: A Method for Identifying DNA *N* ^6^-Methyladenine Sites in the Rice Genome Based on Feature Fusion

**DOI:** 10.3389/fpls.2020.00004

**Published:** 2020-01-31

**Authors:** Qianfei Huang, Jun Zhang, Leyi Wei, Fei Guo, Quan Zou

**Affiliations:** ^1^ College of Intelligence and Computing, Tianjin University, Tianjin, China; ^2^ Rehabilitation Department, Heilongjiang Province Land Reclamation Headquarters General Hospital, Harbin, China; ^3^ Institute of Fundamental and Frontier Sciences, University of Electronic Science and Technology of China, Chengdu, China

**Keywords:** rice, model, fusion, DNA, 6mA

## Abstract

**Motivation:**

The biological function of *N* ^6^-methyladenine DNA (6mA) in plants is largely unknown. Rice is one of the most important crops worldwide and is a model species for molecular and genetic studies. There are few methods for 6mA site recognition in the rice genome, and an effective computational method is needed.

**Results:**

In this paper, we propose a new computational method called 6mA-Pred to identify 6mA sites in the rice genome. 6mA-Pred employs a feature fusion method to combine advantageous features from other methods and thus obtain a new feature to identify 6mA sites. This method achieved an accuracy of 87.27% in the identification of 6mA sites with 10-fold cross-validation and achieved an accuracy of 85.6% in independent test sets.

## Introduction

DNA methylation plays crucial roles in many biological functions, and methylated DNA carries important epigenetic information. The modification of DNA methylation is a heavily researched topic in epigenetic research (Liu et al., 2016). Previously, DNA methylation was thought to comprise cytosine (5-methylcytosine, 5mC) methylation and *N* ^4^-methylcytosine (4mC) methylation (Chen et al., 2017; He et al., 2019; Tang et al., 2019a). However, with the rapid development of sequencing technology, a new type of DNA methylation modification, DNA-6mA methylation, has been identified and has become a heavily researched subject in the field of epigenetics (Xiao et al., 2018). *N* ^6^-methyladenine DNA (6mA) modification is the most prevalent type of DNA modification in prokaryotes. This modification plays important roles in DNA mismatch repair, chromosome replication, cell defense, cell cycle regulation, and transcription (Xu et al., 2017; He et al., 2019). 6mA shows similar properties in eukaryotes and prokaryotes (Hao et al., 2019).

Machine learning methods have overcome many problems in identifying 4mC (Chen et al., 2017) and 5mC modifications. The 6mA modification has become a heavily researched subject, and an increasing number of researchers are using machine learning to identify 6mA sites in the rice genome. The current machine learning algorithms perform notably well in recognizing 6mA sites in the rice genome. Many excellent features and algorithms have been applied to the recognition of 6mA sites. Regarding feature algorithms, nucleotide chemical property, nucleotide frequency (Pan et al., 2017; Yin et al., 2019; Chen et al., 2019a), and mononucleotide binary encoding are often used in the recognition of 6mA sites in the rice genome. These methods all have some properties in common, including the unique representations of nucleotides. This property is also exhibited by our method. Regarding dimensionality reduction algorithms, MRMD (Zou et al., 2016a) performs well and is an excellent feature selection algorithm. Other highly efficient feature selection algorithms have been proposed in bioinformatics classification (Zou et al., 2015; Xu et al., 2017; Zhu et al., 2017; Pan et al., 2018; Wang et al., 2018; Cheng et al., 2018a; Zhu et al., 2018a; Cheng et al., 2018b; Zhu et al., 2018b; Lai et al., 2019; Dao et al., 2019; Yu et al., 2019; Yang et al., 2019; Ren Qi et al., 2019; Tang et al., 2019b). Regarding classification algorithms, an increasing number of classification methods are being used by researchers to identify 6mA sites, such as Random Forest, XGboost, support vector machine (SVM), and gradient boosted decision tree (GBDT). Research has proven that SVM and Random Forest perform better than the other classifier algorithms. In the present study, the performance of SVM was highly stable. A Markov model is used in MM-6mAPred (Pian et al., 2019) to identify 6ma sites and has achieved good results.

There are few computational methods to identify 6mA sites in the rice genome. Proposed methods include i6mA-Pred (Chen et al., 2019a), iDNA6mA-PseKNC (Feng et al., 2019a), MM-6mAPred (Pian et al., 2019), and iDNA6mA-Rice (Hao et al., 2019). i6mA-Pred uses nucleotide chemical property, nucleotide frequency, and SVM to identify 6ma sites. MM-6mAPred adopts a Markov model to identify 6mA sites. iDNA6mA-Rice uses mononucleotide binary encoding and Random Forest to identify 6mA sites. Feature fusion makes use of diverse features to build prediction models and has been successfully and widely applied in bioinformatics (Zhang et al., 2018a; Zhang et al., 2018b; Zhou et al., 2019; Zhang et al., 2019a; Zhang et al., 2019b). In this paper, we propose a feature fusion-based method to identify 6mA sites in the rice genome, in which nucleotide chemical properties, binary encoding, KMER, and Markov features are used to formulate DNA sequences. Our method combines these excellent features by using feature selection algorithms. The proposed model obtained an overall accuracy of 87.27% in identifying 6mA sites.

## Materials and Methods

### Datasets

Two datasets were used in our study. One dataset comprised the same experimental benchmark data used by Chen et al. (Chen et al., 2019a) and has been used to train MM-6mAPred (Cheng et al., 2018b; Pian et al., 2019). This dataset contained 880 positive samples and 880 negative samples. The positive samples were obtained by setting the modification score and CD-HIT. Positive samples can improve the quality of the sequence and reduce redundancy. The second dataset comprised the same experimental benchmark data used to train iDNA6mA-Rice, and it contained 15,400 positive samples and 15,400 negative samples. All of the sequences in these two datasets measured 41 bp in length. All of the negative samples had non-methylated adenosine in the center, and all of the positive samples had a 6mA site in the center.

**Table 1** shows the numbers of positive and negative samples for both datasets. Dataset 1 was mainly used for cross-validation. Dataset 2 was primarily used for independent testing. These two benchmark datasets are available at https://github.com/huangqianfei0916/6ma-rice.

**Table 1:** All datasets.

Datasets	Positive	Negative	Total	Species
Dataset 1	880	880	1,760	Rice
Dataset 2	154,000	154,000	308,000	Rice

### Model Architecture

**Figure 1:**
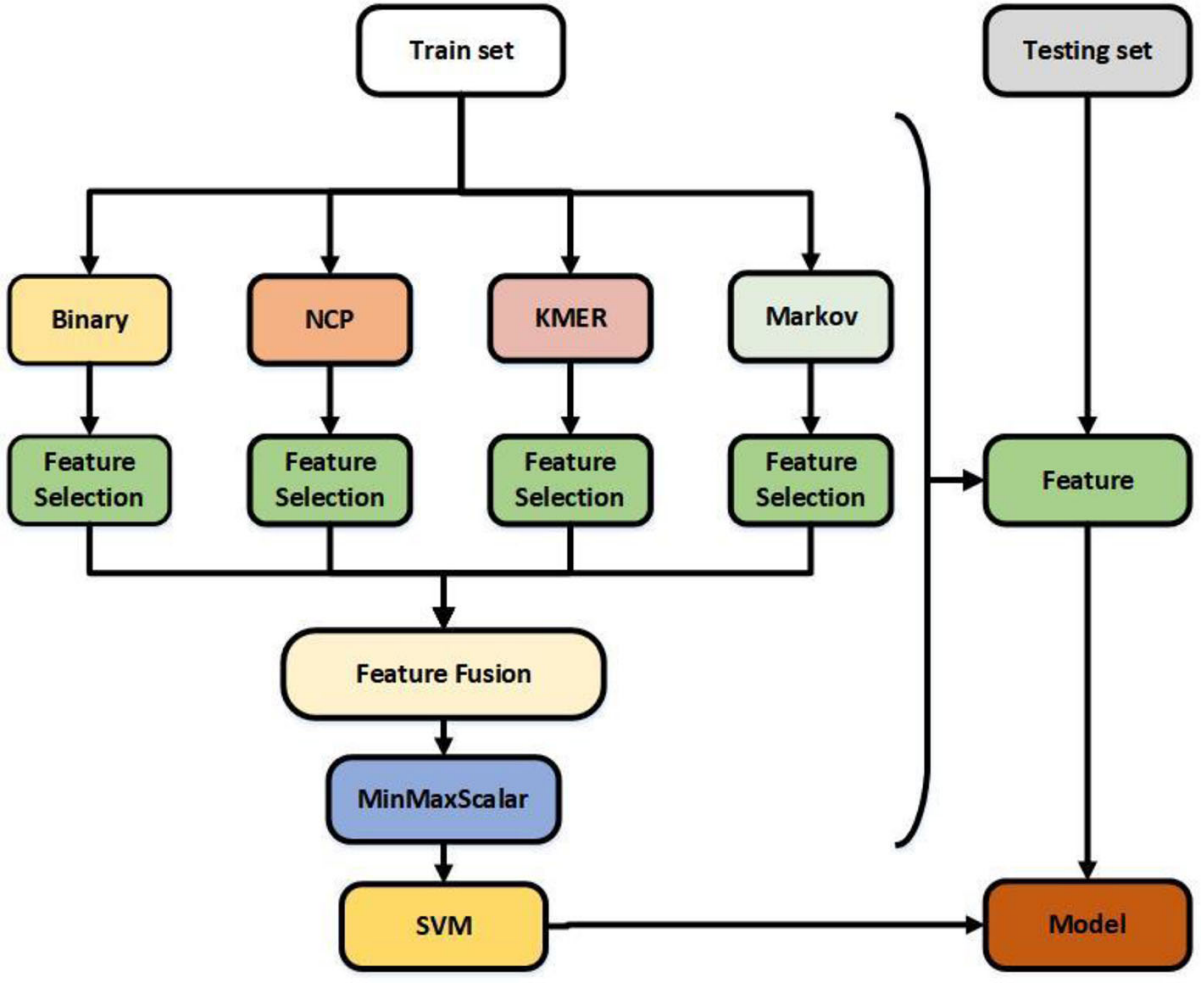
Flowchart showing the construction of this model. The feature selection is selectfrommodel.

Feature extraction plays a crucial role in the construction of the model (Wang et al., 2019). Four feature extraction algorithms were adopted to formulate 6mA samples. Binary encoding, nucleotide chemical property (Xu et al., 2019), KMER, and Markov features were selected from among several feature algorithms, and **Table 1** shows the results of each algorithm. In order to reduce computation and optimize feature vectors, feature selection algorithms were used for each feature. The features after fusion were normalized, and the final feature vectors were the optimal representations of the sequence (Chen et al., 2019b). **Figure 1** illustrates the structure of the model.

Binary encoding and nucleotide chemical property are excellent feature algorithms extracted from iDNA6mA-Rice and i6mA-Pred. Kmer is a useful feature algorithm (Feng et al., 2019b) that we selected based on a large number of experiments. The Markov feature is a new feature extraction algorithm we introduced based on MM-6mAPred. Combining the best features does not necessarily produce the best results; for example, Kmer does not perform well when used alone, but does so when combined with other features. Feature selection solves this problem and reduces the amount of computation. Finally, the best features were obtained by normalization. Regarding the classifier, previous studies have shown that SVM and Random Forest perform better than other classifiers. In this study, the performance of SVM was significantly better than that of Random Forest.

### Binary Encoding

Binary encoding is a simple and effective feature algorithm. This algorithm obtains sequence features by the binary representation of nucleotides (Zou et al., 2019). The binary encoding algorithm converts nucleotides into the following formats:

$$\begin{array}{l} A\to [\begin{array}{cc} 1 & , \end{array}\begin{array}{cc} 0 & , \end{array}\begin{array}{cc} 0 & , \end{array}0]\\ C\to [\begin{array}{cc} 0 & , \end{array}\begin{array}{cc} 1 & , \end{array}\begin{array}{cc} 0 & , \end{array}0]\\ G\to [\begin{array}{cc} 0 & , \end{array}\begin{array}{cc} 0 & , \end{array}\begin{array}{cc} 1 & , \end{array}0]\\ T\to [\begin{array}{cc} 0 & , \end{array}\begin{array}{cc} 0 & , \end{array}\begin{array}{cc} 0 & , \end{array}1] \end{array}$$

This algorithm can be understood as a unique representation of nucleotides and can be considered a one hot encoding algorithm. A random DNA sequence with *m* nucleotides can then be converted into a vector of 4 × *m* features (Hao et al., 2019; Chen et al., 2019c). The representation of nucleotides is not unique, and the representations of A, T, G, and C are interchangeable.

### Nucleotide Chemical Property

DNA is composed of four types of nucleotides: adenine (A), cytosine (C), guanine (G), and cytosine (C). DNA has multiple properties, such as ring structures, functional groups, and hydrogen bonds (Fu et al., 2018; Wei et al., 2018; Xue et al., 2018; Tan et al., 2019a) (He et al., 2019). A and G each contain two rings, whereas C and T contain only one. Regarding secondary structures, A and T form weak hydrogen bonds, whereas C and G form strong hydrogen bonds. Regarding functional groups, A and C compose the amino group, whereas G and T compose the keto group. The feature extraction algorithm can be formulated as follows:

$$\begin{array}{c} a=\{\begin{array}{cc} 1 & n\in \{\begin{array}{cc} A & , \end{array}G\}\\ 0 & \text{others} \end{array}\\ b=\{\begin{array}{cc} 1 & n\in \{\begin{array}{cc} A & , \end{array}T\}\\ 0 & \text{others} \end{array}\\ c=\{\begin{array}{cc} 1 & n\in \{\begin{array}{cc} A & , \end{array}C\}\\ 0 & \text{others} \end{array} \end{array}$$

where *n* represents a nucleotide, which can be converted into the following format:

$$\begin{array}{ll} A\to [\begin{array}{cc} 1 & , \end{array}\begin{array}{cc} 1 & , \end{array}1] & C\to [\begin{array}{cc} 0 & , \end{array}\begin{array}{cc} 0 & , \end{array}1]\\ G\to [\begin{array}{cc} 1 & , \end{array}\begin{array}{cc} 0 & , \end{array}0] & T\to [\begin{array}{cc} 0 & , \end{array}\begin{array}{cc} 1 & , \end{array}0] \end{array}$$

For instance, a DNA sequence “AATCGTA” can be transformed into a vector such as (1,1,1,1,1,1,0,1,0,0,0,1,1,0,0,0,1,0,1,1,1). Nucleotide chemical property has similar properties to binary encoding, both of which can be considered to yield unique representations of nucleotides.

### KMER

Kmer is a highly common feature extraction algorithm and is easy to understand (Liu et al., 2015; He et al., 2018; Su et al., 2018; Zhu et al., 2019). When *k* = 1, Kmer denotes the frequency of the four nucleotides. When *k* = 2, the sequence can be represented by 16 features, i.e., AA, AT, AG, AC, TA, TT, TG, TC, …,CC (Cao et al., 2018). As the value of *k* increases, the dimension of the feature increases; thus, the difficulty of calculation increases. In this study, the *k* value that was employed was 3. Thus, a sequence could be represented as 64 features. We tested the results of *k* from 1 to 4 and chose 3. A *k* equal to 3 will not cause poor results because the features are too sparse. The Kmer (*k* = 3) descriptor can be calculated as follows:

$$p=\frac{t}{L-2}\begin{array}{cc} & t\in \{\mathrm{AAA},\mathrm{AAT},\ldots \ldots ,\mathrm{CCC}\} \end{array}$$

where *L* denotes the length of the sequence and *t* denotes the number of nucleotide occurrences. As the value of *k* increases, the results may improve, but the dimension will increase, causing the amount of calculation to increase. In this study, although Kmer yielded poor results when used alone, the information contained in Kmer was crucial in feature fusion.

### Markov Feature

**Figure 2:**
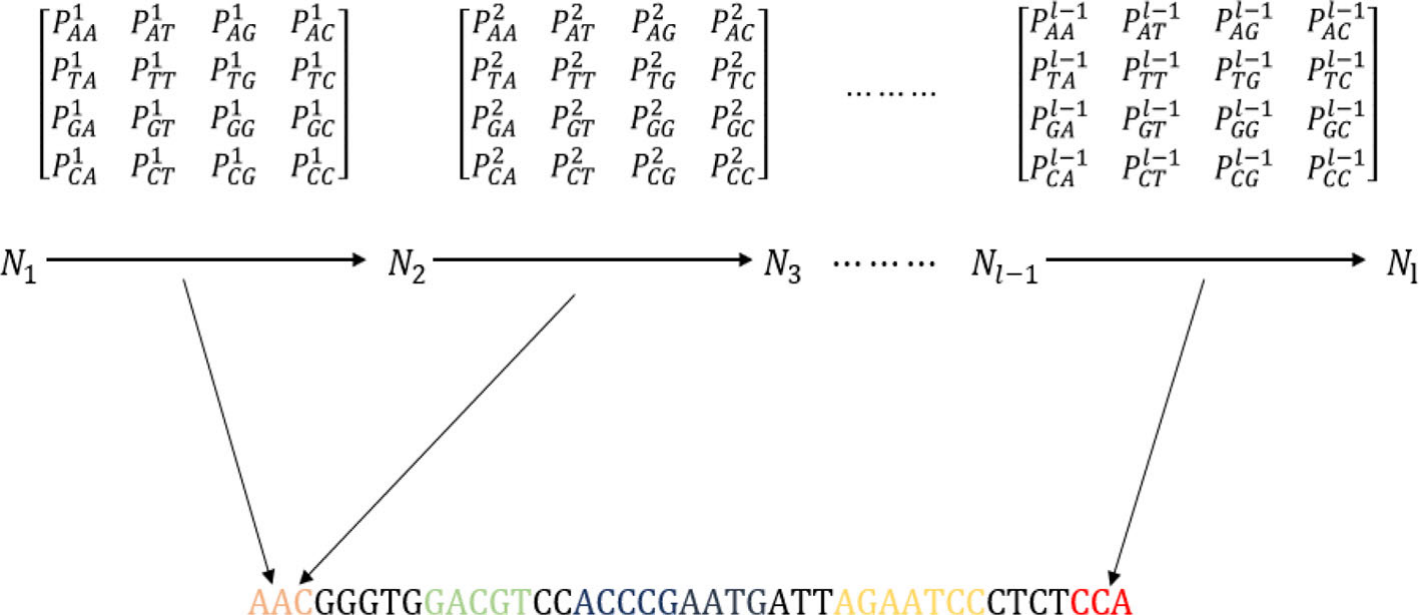
Schematic showing the process of extracting features from the transition probability matrix of the DNA sequence. The sequence “AATACATGGGGTTATGTGCCACCGGTCATAATATCTAGGGT” is used as an example to explain the process.

From MM-6mAPred, we can determine that the Markov chain achieves good performance in recognizing 6ma sites. Therefore, we introduced the Markov chain into DNA sequence analysis to improve the sequence representation. The algorithm constructed the first-order Markov chain (Kemeny and Snell, 1976) for each dataset. Before obtaining the sequence features, the algorithm must calculate the transition probability of the dataset. **Figure 2** shows a schematic of feature extraction from a DNA sequence with the first-order Markov chain. A, T, G, C are equivalent to four states, and *P^i^*_NN_ is the transition probabilities between the *i*th nucleotide and the (*i* + 1)th nucleotide (Nigatu et al., 2017; Pian et al., 2019). Thus, a transition probability matrix is generated between every two nucleotides. A sequence of 41 bp can generate 40 transition probability matrices.

A sequence of 41 bp can be represented as a 40-dimensional vector. We did not use the initial probability because it did not improve the experimental results. The transition probability between two nucleotides is used to represent sequence information. Sequence information between the *i*th nucleotide and the (*i* + 1)th nucleotide is obtained from the *i*th transition probability matrix. Moreover, the features result can be optimized by adjusting the length of the sequence. The sequence contains the transition probability information, and a sequence can be represented by the transition probabilities.

### Performance Evaluation of Different Algorithms

The type of feature algorithm has strong effects on experimental results (Liu et al., 2017; Cheng et al., 2018c; Zheng et al., 2019; Cheng et al., 2019a; Zhang et al., 2019c). After testing many features and classifications, three best-performing classifiers were selected to test the feature descriptors. **Table 2** reports the 10-fold cross-validation results for the classifiers identifying the 6mA sites in dataset 1. Binary encoding, NCP (Chen et al., 2019c), Markov features, and ENAC were selected for feature selection. Experimentation revealed that Kmer is a better choice than ENAC. **Table 2** shows that the results of ENAC are considerably better than those of Kmer, whereas the results from using Kmer fusion are better than those from using ENAC fusion. This finding shows that merging the best-performing features may not be the optimal option. The binary encoding feature algorithm was used in iDNA6mA-Rice, and the NCP feature algorithm was used in i6mA-Pred. Our experimental results were consistent with the results of previous studies. The Markov feature algorithm is a new feature algorithm that we created based on MM-6mAPred. To improve the experimental results and reduce the amount of calculation, the feature selection algorithm is applied for each feature. Feature selection after fusion can also reduce the amount of calculation, but does not achieve as good results. As an alternative approach to feature selection, feature selection can be performed before fusion and again after fusion; however, this approach will result in a few dimensions. Thus, feature selection before fusion is the best approach.

**Table 2:** Performance of different feature descriptors and classifiers.

Feature descriptors	SVM (Acc%)	XGboost (Acc%)	GBDT (Acc %)	Vote (Acc %)
EIIP	63.9	83.9	84.0	83.9
ANF	54.2	60.7	61.1	61.7
BINARY	82.8	84.4	83.6	84.7
DNC	58.4	61.0	59.7	61.2
NCP	82.8	83.3	83.9	84.3
PseEIIP	53.9	66.5	65.3	65.9
TNC	56.8	66.5	65.3	66.0
KMER	53.0	64.2	64.8	65.1
ENAC	73.5	79.4	78.8	79.0
NAC	56.3	55.5	54.6	55.5
CKSNAP	57.2	65.3	65.3	65.8
RCKMER	55.0	62.9	62.3	62.3
MAKOV	83.75	85.17	84.7	85.0

### Support Vector Machine

SVM is a widely used machine learning algorithm (Ding and Li, 2015; Li et al., 2015; Zeng et al., 2017; Ding et al., 2017a; Zhang et al., 2019; Tan et al., 2019a) and was used in this study to identify 6mA sites in the rice genome. SVM is also widely used in bioinformatics fields (Zou et al., 2016b; Wang et al., 2018; Wei et al., 2018; Xiong et al., 2018; Zeng et al., 2018a; Xu et al., 2018a; Xu et al., 2018b; Xu et al., 2018c; Li et al., 2019). Our experiments showed that SVM was more suitable for the purposes of the present study than were the other algorithms. We used the libsvm package available at http://www.csie.ntu.edu.tw/~cjlin/libsvm/. The radial basis kernel function (RBF) was used to obtain the classification hyperplane. The two main parameters of SVM, *C* and gamma, were optimized by grid search. The optimization ranges about *C* and gamma were (2^−5^,2^5^) and (2^−5^,2^5^), respectively, and the values of *C* and gamma were 1.0 and 0.125, respectively. In this study, SVM performed better than the other classifiers.

### Feature Selection

Feature selection algorithms are widely used in machine learning (Liu X. et al., 2019; Zeng et al., 2019a; Zeng et al., 2019b), and feature selection is necessary with our method. Feature selection removes redundant and uncorrelated information from the sequence and increases computational speed. In this study, we chose the selectfrommodel module of sklearn and the classifier XGboost (Chen and Guestrin, 2016; Zhou et al., 2019). Feature selection can optimize features and reduce the number of calculations. The results of our experiments proved that feature selection can improve results and reduce computation. Feature selection was able to identify the better features, and XGboost was the best-performing classifier. We investigated other feature selection methods, but did not obtain high-quality results. Feature selection can be performed in three ways: before fusion, after fusion, and both before and after fusion. The experimental results showed that before fusion is the best approach.

### Performance Evaluation

It is important to evaluate the results of a new model, and several evaluation metrics are available. Sensitivity (Sn), specificity (Sp), accuracy (Acc), and Mathew’s correlation coefficient (MCC) are often used to evaluate the quality of a model in machine learning (Liu B. et al., 2019; Cheng et al., 2012; Cheng et al., 2016; Ding et al., 2016b; Mariani et al., 2017; Ding et al., 2017; Xu et al., 2017; Wei et al., 2017a; Wei et al., 2017b; Hu et al., 2018; Zhang et al., 2018c; Ding et al., 2019; Shan et al., 2019; Xu et al., 2019; Tan et al., 2019b; Cheng et al., 2019b). These metrics are formulated as follows:

$$\begin{array}{c} \mathrm{Sn}=\frac{\mathrm{TP}}{\mathrm{TP}+\mathrm{FN}}\\ \mathrm{Sp}=\frac{\mathrm{TN}}{\mathrm{TN}+\mathrm{FP}}\\ \mathrm{Acc}=\frac{\mathrm{TP}+\mathrm{TN}}{\mathrm{TP}+\mathrm{TN}+\mathrm{FP}+\mathrm{FN}}\\ \mathrm{MCC}=\frac{\mathrm{TP}*\mathrm{TN}-\mathrm{FP}*\mathrm{FN}}{\sqrt{\left(\mathrm{TP}+\mathrm{FP}\right)*\left(\mathrm{TP}+\mathrm{FN}\right)*\left(\mathrm{TN}+\mathrm{FP}\right)*\left(\mathrm{TN}+\mathrm{FN}\right)}} \end{array}$$

These metrics are commonly used in machine learning. TP, TN, FP, and FN denote true positive, true negative, false positive, and false negative, respectively. The above mathematical expressions clearly describe the meanings of the four metrics. In model evaluation methods, independent dataset testing and cross-validation are often used to evaluate the prediction ability of the model. In this study, dataset 1 was mainly used for cross-validation and training, and dataset 2 was mainly used for independent testing. In the independent test experiment, dataset 1 was used for training and dataset 2 was used for testing.

In addition to the above metrics, area under the ROC curve (AUC) and receiver operating characteristic (ROC) were also used to evaluate model quality.

## Results and Discussion

### Analysis of the Algorithms

**Figure 3:**
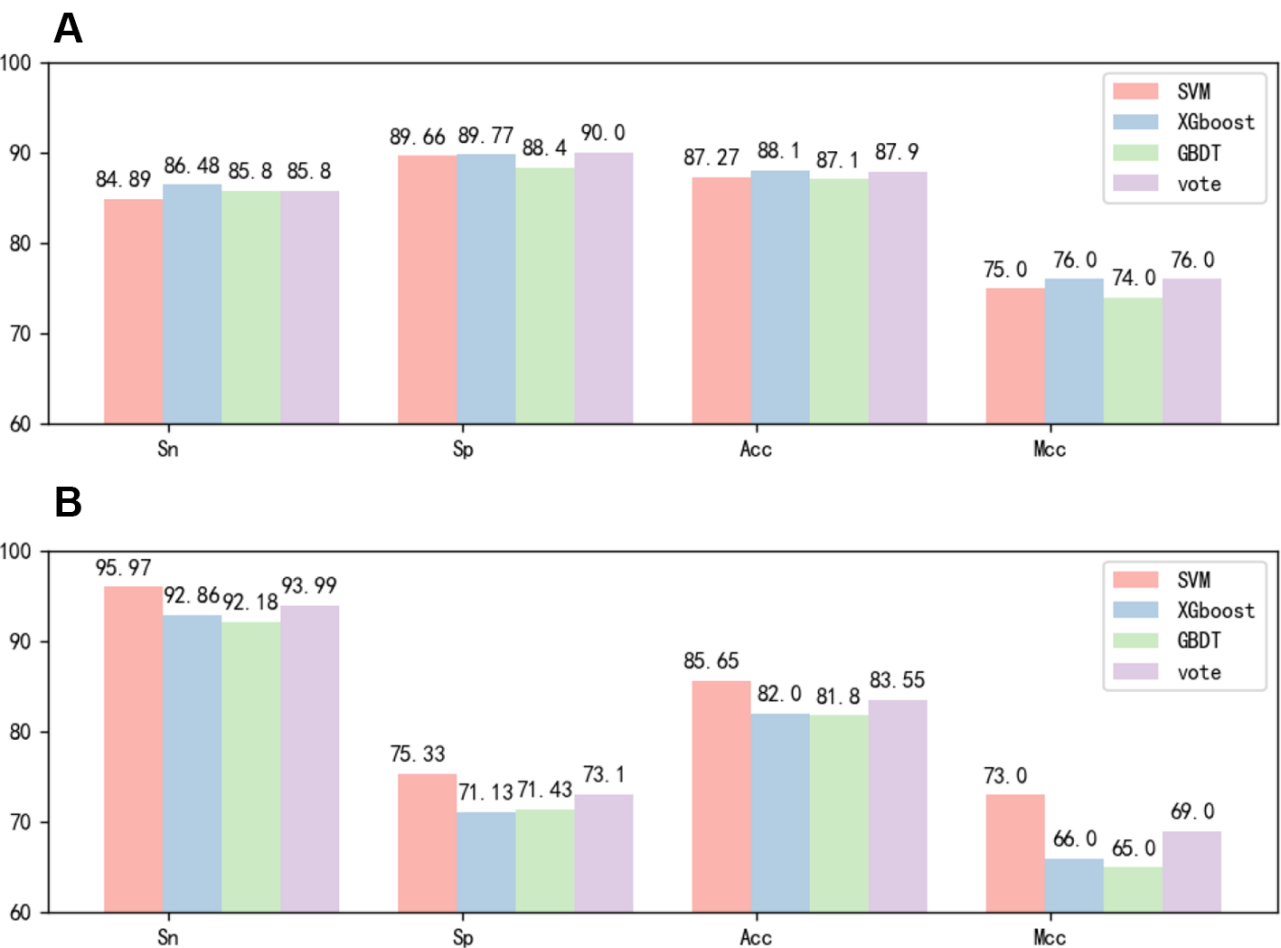
**(A)** Tenfold cross-validation performance of different classifiers based on dataset 1. **(B)** Independent test performance of different classifiers based on dataset 1 and dataset 2.

After the feature fusion, we tested the feature using voting techniques and three different classifiers with 10-fold cross-validation and independent test experiments. The 10-fold cross-validation results of the different methods in identifying 6mA sites by using the benchmark dataset 1 are reported in **Figure 3A**. The independent test results of the different methods in identifying 6mA sites by using the benchmark dataset 2 are reported in **Figure 3B**. **Figure 3A** shows that the test results of the three classifiers were highly similar. However, **Figure 3B** shows that SVM performed significantly better than the other classifiers. Based on the experimental results, we chose the SVM classifier in this study.

Before feature extraction, we conducted a simple optimization of the sequence length. In addition, we tested the original sequence and the optimal sequence using our method. The experimental results are reported in **Table 3**. As depicted in the table, feature selection is an excellent choice; the results of the best sequence were considerably better than those of the original sequence. The results revealed no significant improvement; however, reducing the length of the sequence reduces the amount of calculation.

**Table 3:** Cross-validation performance of different methods based on dataset 1.

Method	Sn	Sp	Acc	Mcc
Best sequence—no fs	81.81	88.30	85.1	0.702
Original sequence—no fs	84.20	84.77	84.49	0.690
Best sequence—fs	84.89	89.66	87.27	0.746
Oraigin sequence—fs	85.0	89.20	87.10	0.742

**Figure 4:**
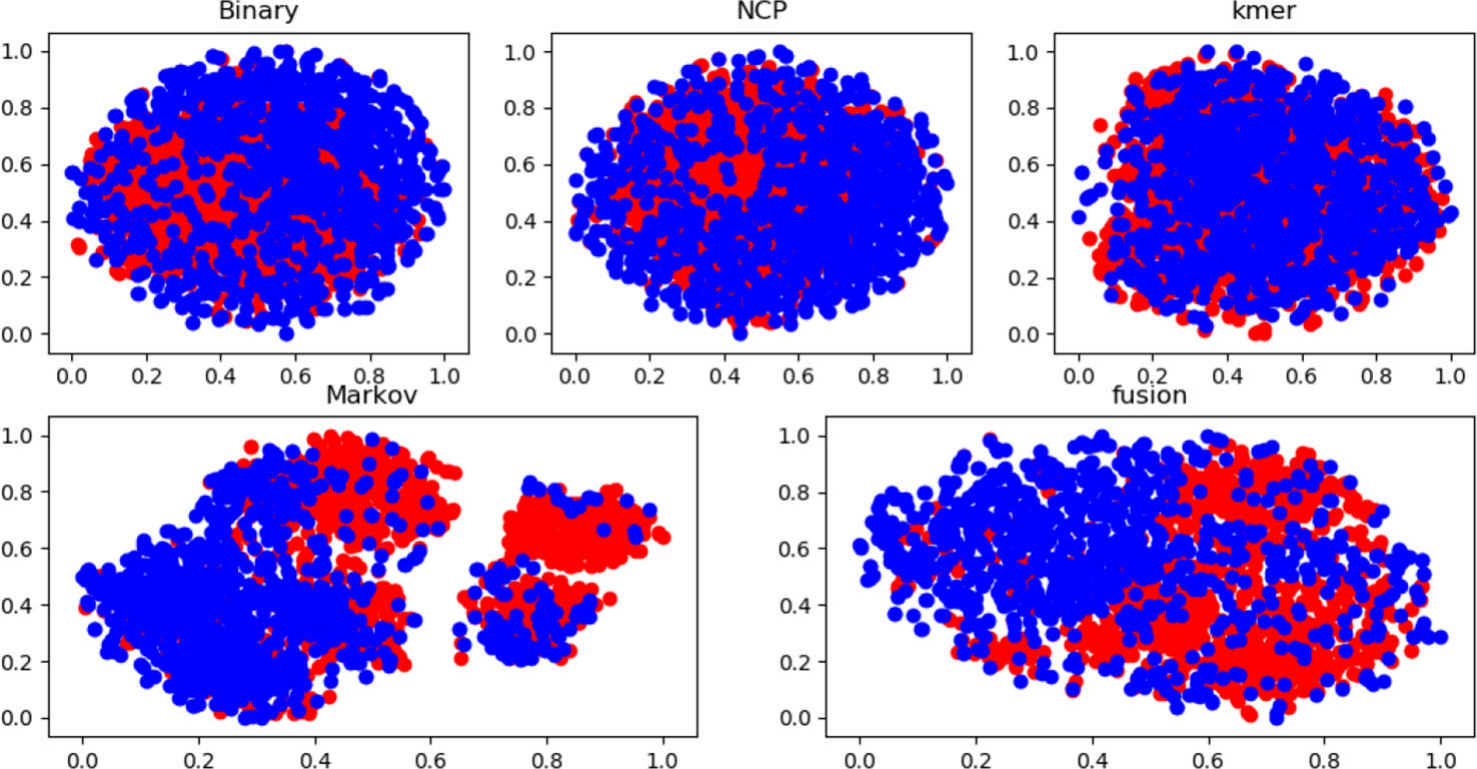
Feature distribution of different feature methods based on dataset 1.

**Figure 5:**
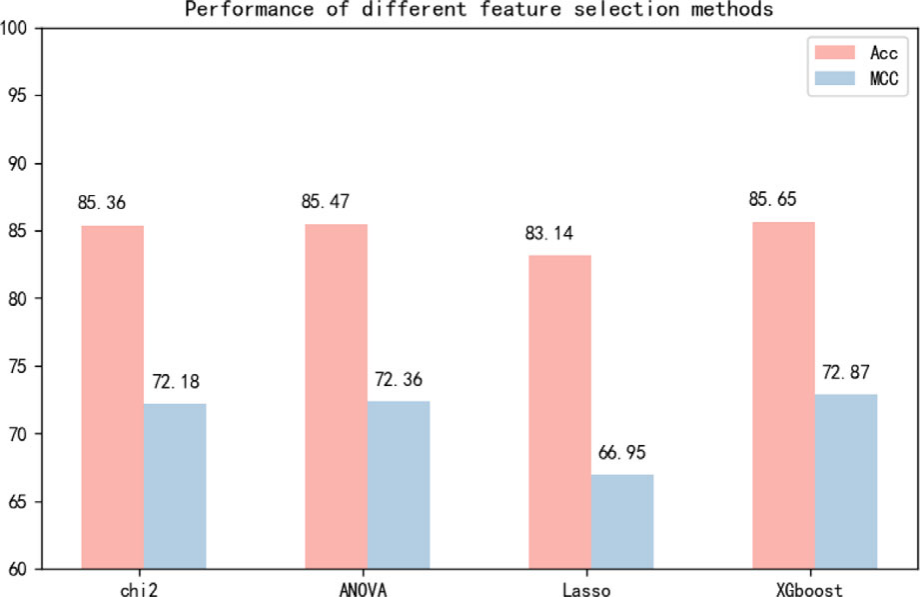
Independent test performance of different feature selection methods based on dataset 1 and dataset 2.

Many experiments have been conducted regarding the selection of feature algorithms and classifiers. Our experiments revealed that binary encoding, NCP, and the Markov feature were effective, and previous studies have shown that they yield good results when used alone. We visualize the features by reducing the dimensionality, and **Figure 4** reports the distribution of each feature method. Therefore, we combined these excellent features to improve representation. In the selection of feature selection methods, we tested several widely used methods, and the experimental results are shown in **Figure 5**. To further optimize the features, we applied MinMaxScaler to the features after fusion. The differences between **Figures 3A, B** indicate that the SVM was highly robust. Similarly, the model obtained by learning the optimized features with SVM was highly powerful. The method can be applied to computational intelligence techniques, such as neural networks (Chen et al., 2016; Song et al., 2018; Cabarle et al., 2019; Hong et al., 2019; Zhong et al., 2019; Zhou et al., 2019b; Wang et al., 2019b), evolutionary algorithms (Xu et al., 2019; Xu et al., 2019; Zeng et al., 2019b), and unsupervised learning (Zeng et al., 2018b; Zou et al., 2019), in future research.

### Comparisons With Other Methods

To prove that our method is superior to other methods, we compared it with MM-6mAPred and i6mA-Pred, which are two excellent methods. i6mA-Pred uses nucleotide chemical property and nucleotide frequency as the features and reduces the dimensions by MRMD (Zou et al., 2016a). This approach then uses SVM to identify the 6mA sites in the rice genome. MM-6mAPred identifies 6mA sites based on the Markov model. The i6mA-Pred method is easy to understand, and its performance is good. The MM-6mA-Pred method is novel and has achieved good results. MM-6mA-Pred constructs multiple transition probability matrices for positive and negative samples. The new sample calculates the product of the transition probabilities in the two sets of transition probability matrices. The sample makes predictions based on the ratio of the two products. In addition, MM-6mA-Pred optimizes the length of the sequence to achieve optimal results, and similar operations are performed in our method. In general, the two methods yield effective models from different perspectives.

To improve experimental results, the main features of both methods are included in our method. In this study, we used feature selection and sequence length optimization, and we used 10-fold cross-validation and independent testing to evaluate the method. To conduct comparative tests, we reproduced the MM-6mAPred model with python3 and used the metrics we used previously for evaluation. In the cross-validation experiment, we performed 10-fold cross-validation based on dataset 1. The MM-6mAPred model that we reproduced obtained an accuracy of 84.7%, which is lower than the 89.7% reported in the paper in which the model is proposed. In the independent test experiment, dataset 1 was used for training and dataset 2 was used for testing. The model that we reproduced with python3 and the model implemented with MATLAB by the authors of the source paper yielded consistent results. The independent testing experiments revealed that the accuracy of MM-6mAPred was only 83.06%, whereas our method achieved 85.65% accuracy. Similar tests were performed with i6mA-Pred, and the results are reported in **Tables 4** and **5**. The experimental results show that our method is superior to other methods. In addition, the results obtained with MM-6mAPred were better than the results obtained with i6mA-Pred. Our reproduced MM-6mAPred code has been deposited on GitHub at https://github.com/huangqianfei0916/Markov.

**Table 4:** Cross-validation performance of different methods based on dataset 1.

Method	Sn	Sp	Acc	Mcc
Our method	84.89	89.66	87.27	0.746
MM-6mAPred	84.31	85.22	84.77	0.695
i6mA-Pred	82.95	83.30	83.13	0.662

**Table 5:** Independent test performance of different methods based on dataset 1 and dataset 2.

Method	Sn	Sp	Acc	Mcc
Our method	95.97	75.33	85.65	0.73
MM-6mAPred	95.81	70.30	83.06	0.68
I6mA-Pred	94.24	66.59	80.42	0.63

**Figure 6:**
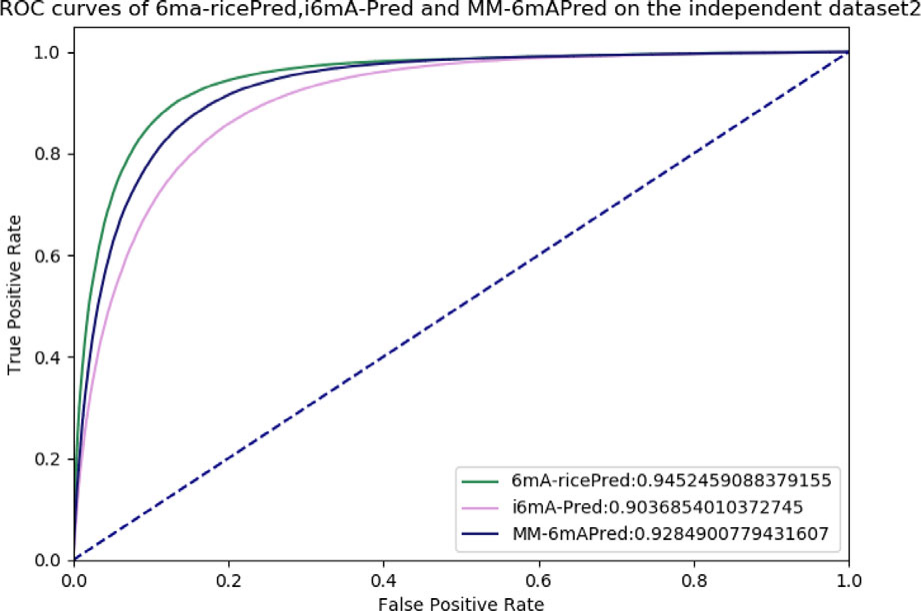
Receiver operating characteristic (ROC) curves of 6ma-ricePred, MM-6mAPred, and i6mA-Pred.

Allowing further comparisons of these methods, ROC and AUC are shown in **Figure 6**. The area under the curve values (AUCs) of 6mA-ricePred, MM-6mAPred, and i6mA-Pred were 0.945, 0.928, and 0.904, respectively. **Figure 6** shows that our method performed better than the other two methods.

## Conclusion

Accuracy in identifying DNA *N* ^6^-methyladenine sites is highly important. The chemical properties of the nucleotides and the Markov model were used in i6mA-Pred and MM-6mAPred, respectively, and achieved good results. Our method, which is based on feature fusion, achieved better results than these previous methods in identifying 6mA sites in the rice genome. Our method obtains a more powerful model by combining multiple effective methods. These experiments proved that the proposed method is superior to other methods, and it integrates the main features of previous methods.

We found that in addition to machine learning, the attention mechanism of deep learning can be applied to the recognition of 6mA sites in the rice genome. Amplifying the information of the 6mA sites by assigning attention can improve the recognition rate. The deep learning attention mechanism can be introduced into machine learning by, for example, multiplying different features by different weights and dynamically adjusting the weights according to the importance of the features.

## Data Availability Statement

Publicly available datasets were analyzed in this study. This data can be found here: https://github.com/huangqianfei0916/6ma-rice/tree/master/dataset.

## Author Contributions

QH and JZ provided the data and did a lot of experiments. QZ and FG guided and modified the paper. LW provides a lot of good advice.

## Funding

The work was supported by the National Natural Science Foundation of China (no. 91935302, no. 61922020, no. 61771331) and the Scientific Research Foundation in Shenzhen (JCYJ20180306172207178).

## Conflict of Interest

The authors declare that the research was conducted in the absence of any commercial or financial relationships that could be construed as a potential conflict of interest.
